# Causes of Hospitalization among End-Stage Kidney Disease Cohort before and after Hemodialysis

**DOI:** 10.3390/ijerph191610253

**Published:** 2022-08-18

**Authors:** Hsiu-Lan Li, Pei-Hui Tai, Yi-Ting Hwang, Shih-Wei Lin, Li-Ching Lan

**Affiliations:** 1 Department of Nursing, Yunghe Cardinal Tien Hospital, New Taipei City 23148, Taiwan; 2 Graduate Institute of Management, Chang Gung University, Taoyuan City 33302, Taiwan; 3 Medical Quality Control Room, En Chu Kong Hospital, New Taipei City 23148, Taiwan; 4 Department of Statistics, National Taipei University, New Taipei City 23148, Taiwan; 5 Department of Information Management, Chang Gung University, Taoyuan City 33302, Taiwan; 6 Department of Emergency Medicine, Keelung Chang Gung Memorial Hospital, Keelung City 20641, Taiwan; 7 Department of Industrial Engineering and Management, Ming Chi University of Technology, New Taipei City 23148, Taiwan; 8 Department of Nursing, En Chu Kong Hospital, New Taipei City 23148, Taiwan

**Keywords:** end-stage kidney disease, end-stage renal disease, hospitalizations, chronic hemodialysis

## Abstract

Patients with end-stage kidney disease (ESKD) have a greater risk of comorbidities, including diabetes and anemia, and have higher hospital admission rates than patients with other diseases. The cause of hospital admissions is associated with ESKD prognosis. This retrospective cohort study involved patients with ESKD who received hemodialysis and investigated whether the cause of hospital admission changed before versus after they started hemodialysis. This study recruited 592 patients with ESKD who received hemodialysis at any period between January 2005 and November 2017 and had been assigned the International Classification of Diseases Ninth Revision Clinical Modification (ICD-9-CM) code for ESKD. The patients’ demographic data and hospitalization status one year before and two years after they received hemodialysis were analyzed. A McNemar test was conducted to analyze the diagnostic changes from before to after hemodialysis in patients with ESKD. The study’s sample of patients with ESKD comprised more women (51.86%) than men and had an average age of 67.15 years. The numbers of patients admitted to the hospital for the following conditions all decreased significantly after they received hemodialysis: type 2 (non-insulin-dependent and adult-onset) diabetes; native atherosclerosis; urinary tract infection; gastric ulcer without mention of hemorrhage, perforation, or obstruction; pneumonia; reflux esophagitis; duodenal ulcer without mention of hemorrhage, perforation, or obstruction; and bacteremia. Most patients exhibited one or more of the following comorbidities: diabetes (*n* = 407, 68.75%), hypertension (*n* = 491, 82.94%), congestive heart failure (*n* = 161, 27.20%), ischemic heart disease (*n* = 125, 21.11%), cerebrovascular accident (*n* = 93, 15.71%), and gout (*n* = 96, 16.22%). An analysis of variance (ANOVA) indicated that changes in the ICD-9-CM codes for native atherosclerosis, urinary tract infection, pneumonia, and hyperkalemia were associated with age. Patients who developed pneumonia before or after they received hemodialysis tended to be older (range: 69–70 years old). This study investigated the causes of hospital admission among patients with ESKD one year before and two years after they received hemodialysis. This study’s results revealed hypertension to be the most common comorbidity. Regarding the cause of admission, pneumonia was more prevalent in older than in younger patients. Moreover, changes in the ICD-9-CM codes of native atherosclerosis, urinary tract infection, pneumonia, and hyperkalemia were significantly correlated with age. Therefore, when administering comprehensive nursing care and treatment for ESKD, clinicians should not only focus on comorbidities but also consider factors (e.g., age) that can affect patient prognosis.

## 1. Introduction

End-stage kidney disease (ESKD) has been a global public health problem over the past few decades [1,2], and research teams around the globe have investigated ESKD’s causes and effects [3,4]. Taiwan has higher incidence and prevalence rates of ESKD than developed countries. Compared with other diseases, ESKD results in a relatively large healthcare burden and a high hospital admission rate [5,6]. According to the 2016 Annual Data Report published by the United States Renal Data System, admissions and readmissions to hospitals constituted the major burden to patients with ESKD, where the average patient with ESKD is admitted to the hospital twice a year [7]. The high medical cost of ESKD treatment has been an increasing burden on Taiwan’s healthcare system. According to the National Health Research Institutes’ 2018 Annual Report on Kidney Disease in Taiwan, the 2016 incidence and prevalence rates of ESKD requiring dialysis were 493 and 3392 people per million population, respectively; these figures equate to an annual increase of 1.9% over the previous three years. The admission rate per 1000 dialysis patients increased from 923 admissions in 2000 to 1015 admissions in 2016, indicating the increase in admission rate among patients receiving dialysis [8], and this increasing trend was primarily attributable to the admission of patients with ESKD. Accordingly, reducing the admission rate and monitoring dialysis quality among patients with ESKD have become urgent issues for health care policy makers in Taiwan and abroad.

Compared with other diseases, ESKD has a higher mortality rate and involves a greater utilization of outpatient, emergency, and inpatient services due to its more complex clinical comorbidities (e.g., diabetes, heart diseases, anemia, cardiovascular diseases (stroke and peripheral vascular disease), lower albumin levels, and lower hematocrits) and higher risks of stroke, myocardial infarction, congestive heart failure, infectious disease, and sepsis [3,9,10]. Patients with ESKD are most burdened by admissions and readmissions, which become more frequent and severe as the disease progresses [5,11]. Observational studies conducted by a single medical institution or health maintenance organization have also demonstrated that patients with ESKD have a high burden of comorbidities [12]. In Taiwan, a 2016 survey of the prevalence rate of comorbidities in dialysis patients revealed that the top three comorbidities were as follows (percentage of all dialysis patients with the comorbidity written in parentheses): hypertension (83.3%), cardiovascular diseases (57.1%), and diabetes (50.4%).

The early determination of the risk of hospitalization among patients with ESKD potentially reduces the likelihood of hospitalization [13]. Studies have indicated that in patients with ESKD, (1) patient condition and hospitalization rate are positively correlated, and (2) the frequency and cause of hospital admissions affect ESKD prognosis [7,14]. Studies on patients with ESKD have mostly focused on admission trends and the death rate in the first year of dialysis; these studies have rarely compared patient status before versus after undergoing dialysis Metcalfe, Khan, Prescott, Simpson, and Macleod [5]. This study conducted a retrospective cohort analysis on all patients with ESKD who received hemodialysis and investigated whether the cause of hospital admission changed before versus after they started hemodialysis.

## 2. Materials and Methods

### 2.1. Ethics Statement

This study was approved by an institutional review board (reference number: ECKIRB1061202), and all experimental methods in this study conformed to the Declaration of Helsinki. Considering the low-risk nature of this study and its retrospective research design, the institutional review board waived the requirement for informed consent from patients.

### 2.2. Data Collection

**Figure 1:**
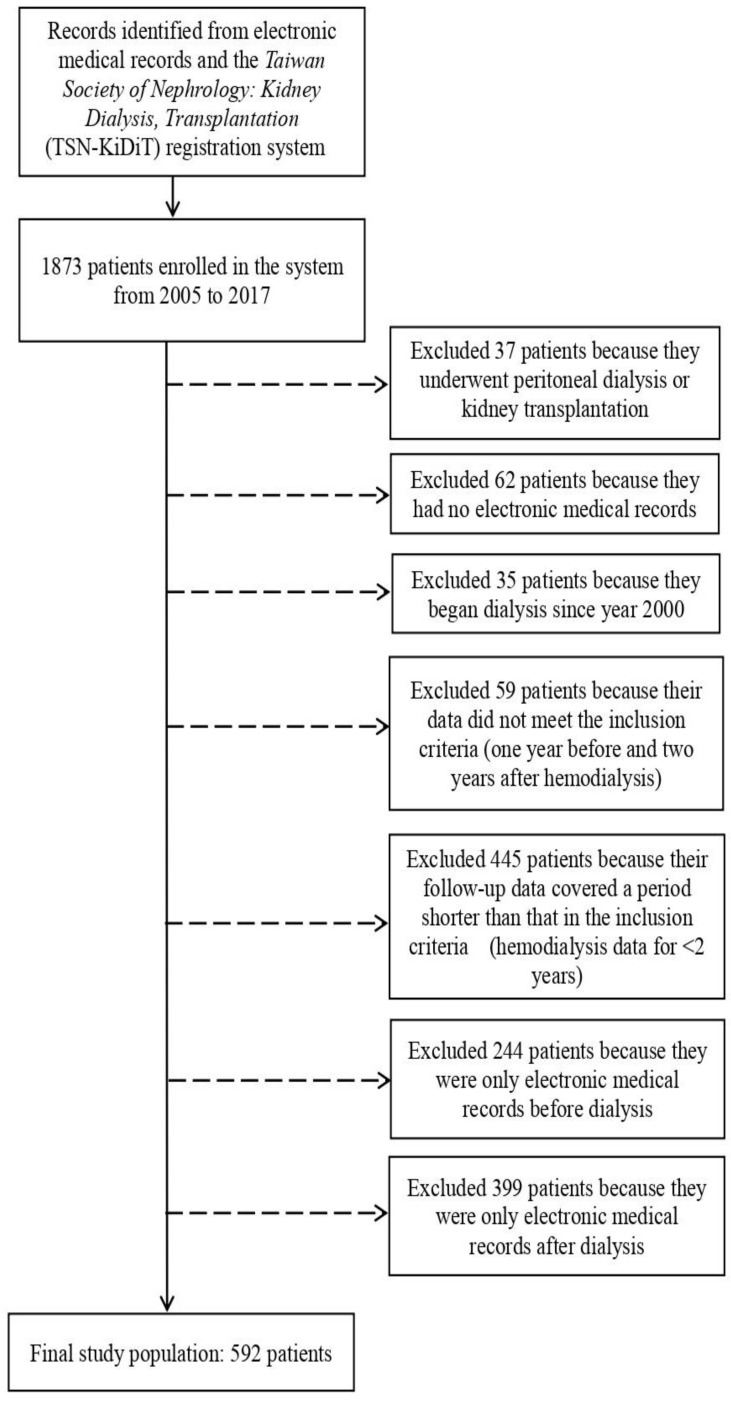
Patient flow chart of this study.

In this study, data were obtained from the electronic medical records of patients with ESKD and from the Taiwan Society of Nephrology: Kidney Dialysis, Transplantation (TSN-KiDiT) registration system. The TSN-KiDiT has been receiving reports from dialysis centers in Taiwan since 1997. It integrates the medical records from Taiwanese hospitals and dialysis clinics of all patients with ESKD who have received chronic hemodialysis. The TSN-KiDiT includes demographic statistics, related diseases, the date of first dialysis treatment, the type of dialysis, residual renal function, and laboratory data of all patients receiving dialysis in Taiwan. This study collected annual reports of dialysis facilities, including dialysis dose, treatment quality, laboratory data, and clinical results. Because the TSN-KiDiT facilitates access to existing patient data systems, this study collected all the medical records of patients with ESKD. The study was conducted at a teaching hospital in Taiwan. This study aimed to investigate whether the patients were assigned different International Classification of Diseases Ninth Revision Clinical Modification (ICD-9-CM) codes 403.1, 403.11, 403.91, 404.02, 404.03, 404.12, 404.13, 404.92, 404.93, 585, and 586 at 1 year before versus 2 years after they received hemodialysis. Patients were included if they satisfied the following criteria: assigned the ICD-9-CM code for ESKD, aged 20 years or older, received hemodialysis at this hospital during any period between January 2005 and November 2017 and had been hospitalized only in the teaching hospital. Accordingly, this study excluded 37 patients who had either been receiving peritoneal dialysis for a long time or had received a kidney transplant; 62 patients who had no record of hospitalization; 35 patients who had started their hemodialysis in 2000; 59 patients whose data did not satisfy the inclusion criteria (1 year before and 2 years after hemodialysis); 445 patients whose follow-up data were for a period shorter than that in the inclusion criteria (hemodialysis data for <2 years); 244 patients who had a hospitalization record only for before their hemodialysis; and 399 patients (1) who had a hospitalization record only for after their hemodialysis, (2) who had a period of less than 2 years when their hemodialysis began and ended, and (3) who had been transferred between hospitals. Finally, 592 patients were recruited as participants (Figure 1).

All diagnoses of ESKD involved a nephrologist and were based on the 2012 Kidney Disease Improving Global Outcome guidelines; the nephrologist evaluated the disease condition, confirmed the diagnosis, and determined the stage of chronic kidney disease according to the cause, glomerular filtration rate (GFR), and albuminuria category (CGA) [15]. All recruited patients in the present study were in the fifth stage of chronic kidney disease, having a GFR < 15 mL/min/1.73 m^2^ [16].

This study collected the demographic data of patients with ESKD and their hospitalization data for one year before and two years after they received hemodialysis. The demographic and clinical data comprised age, sex, primary disease category, comorbidities, and reason for hospitalization. ICD-9-CM codes were used to record all primary and secondary diagnoses of patients with ESKD in the hospitalization data files. The recruited patients were assigned a total of 2995 ICD-9-CM diagnosis codes, 1% of which (100 codes) were retained in the study for analysis. Hemodialysis quality indices were established according to the On-Site Inspection Standards for Hemodialysis and Peritoneal Dialysis Practices and the clinical indices in the dialysis database published by the Taiwan Society of Nephrology [8,17]. Six comorbidities (ICD-9-CM) were analyzed in this study: diabetes, hypertension, congestive heart failure, ischemic heart disease, cerebrovascular accident, and gout [8,17,18,19].

## 3. Statistical Analysis

The McNemar test employed in this study is a simple and common method used for binary matched-pairs data. This test can be used in longitudinal observational research, and it involves a 2 × 2 contingency table for testing the correlation between randomly paired variables [20,21,22]. This study first used the McNemar test to evaluate how the 100 primary and secondary diagnoses changed before versus after the patients received hemodialysis. The significance level was set at 0.05. Consulting the literature and considering which diagnoses significantly differed between before and after hemodialysis, this study selected 11 reasons for hospitalization: diabetes; essential hypertension; anemia; native atherosclerosis; urinary tract infection; gastric ulcer without mention of hemorrhage, perforation, or obstruction (hereafter gastric ulcer); pneumonia; reflux esophagitis; duodenal ulcer without mention of hemorrhage, perforation, or obstruction (hereafter duodenal ulcer); hyperkalemia; and bacteremia. The ICD-9-CM codes of these conditions were then used in a chi-square test to determine the association of the diagnosis changes with the six comorbidities and demographic variables [8,23,24,25,26]. An analysis of variance (ANOVA) was then conducted to investigate the associations of age with those ICD-9-CM codes that exhibited significant differences before versus after hemodialysis. SAS for Windows version 9.4 (SAS Institute, Cary, NC, USA) was used to process and analyze the data.

## 4. Results

Table 1 presents the distributions of the demographic data. A retrospective analysis was conducted on 592 patients who were assigned the ICD-9-CM code for ESKD at any time between January 2005 and November 2017. These patients comprised more women (51.86%) than men and had an average age of 67.15 years old (range: 24–94 years old, standard deviation = 12.29 years). The patients were segmented by when their hemodialysis began: ≤2005 (*n* = 97, 16.39%), 2006–2010 (*n* = 202, 34.12%), 2011–2013 (*n* = 133, 22.47%), and >2013 (*n* = 160, 27.03%). Three major primary disease categories were determined, namely “kidney disease”, “systemic disease”, and “others”. The systemic disease category accounted for the largest proportion of patients (*n* = 432, 72.97%). Most patients developed one or more of the following comorbidities: diabetes (*n* = 407, 68.75%), hypertension (*n* = 491, 82.94%), congestive heart failure (*n* = 161, 27.20%), ischemic heart disease (*n* = 125, 21.11%), cerebrovascular accident (*n* = 93, 15.71%), and gout (*n* = 96, 16.22%).

Table 2 presents the relationship between diagnoses (ICD-9-CM codes) and patients’ hospitalization status before and after they received hemodialysis. The McNemar test was used to examine how the 100 ICD-9-CM diagnosis codes [1,8,22,23] changed before versus after hemodialysis. This study analyzed only 11 conditions that exhibited a statistical difference before versus after the treatment as follows: type 2 (non-insulin-dependent and adult-onset) diabetes (hereafter type 2 diabetes), essential hypertension, anemia, native atherosclerosis, urinary tract infection, gastric ulcer, pneumonia, reflux esophagitis, duodenal ulcer, hyperkalemia, and bacteremia. With regard to the causes of admission, the probabilities of being admitted for the following conditions decreased significantly after the patients received hemodialysis: type 2 diabetes, native atherosclerosis, urinary tract infection, gastric ulcer, pneumonia, reflux esophagitis, duodenal ulcer, and bacteremia. Comparing the numbers of patients admitted to the hospital for the 11 conditions before and after patients received hemodialysis, the result revealed a significant decrease from 65 admissions for type 2 diabetes (10.98%) before hemodialysis to 41 admissions (6.93%) after hemodialysis (*p* = 0.020); from 74 (12.50%) to 28 (4.73%) for native atherosclerosis (*p* < 0.001); from 90 (15.20%) to 58 (9.80%) for urinary tract infection (*p* = 0.009); from 91 (15.37%) to 34 (5.74%) for gastric ulcer (*p* < 0.001); from 113 (19.09%) to 58 (9.80%) for pneumonia (*p* < 0.001); from 68 (11.49%) to 40 (6.76%) for reflux esophagitis (*p* = 0.007); from 60 (10.14%) to 33 (5.57%) for duodenal ulcer (*p* = 0.005); and from 52 (8.78%) to 10 (1.69%) for bacteremia (*p* < 0.001). However, the probabilities of admission for essential hypertension, anemia, and hyperkalemia increased significantly after hemodialysis. The result revealed a significant increase in the number of admissions from 20 (3.38%) before hemodialysis to 112 (18.92%) after hemodialysis for essential hypertension (*p* < 0.001); from 76 (12.84%) to 124 (20.95%) for anemia (*p* < 0.001); and from 41 (6.93%) to 69 (11.66%) for hyperkalemia (*p* = 0.008).

Table 3 presents the relationship between age and the 11 ICD-9-CM codes that had exhibited significant differences before versus after hemodialysis. According to the ANOVA model, age was correlated with status changes in native atherosclerosis, urinary tract infection, pneumonia, and hyperkalemia. Younger patients were less likely to be admitted to the hospital for native atherosclerosis, urinary tract infection, and pneumonia before and after hemodialysis. Patients with hyperkalemia aged between 65 and 66 years and those who received hemodialysis were more likely to be admitted to the hospital for hyperkalemia (average age: 64 years old). Patients who had experienced urinary tract infection and hyperkalemia before they received hemodialysis tended to be older (average age: 70 years old). Older patients aged approximately 69–70 years old were more likely to develop pneumonia either before or after hemodialysis. Older patients were also more likely to have a urinary tract infection relative to their younger counterparts.

This study conducted a chi-square test to analyze the correlation between the six comorbidities and the changes in hospitalization diagnosis (ICD-9-CM) before versus after hemodialysis. Among patients diagnosed with type 2 diabetes, the probabilities of admission for diabetes, hypertension, and gout increased significantly after hemodialysis. Specifically, the number of admissions increased from 38 (9.34%) before hemodialysis to 50 (12.29%) after hemodialysis for diabetes (*p* < 0.001); from 35 (7.13%) to 52 (10.59%) for hypertension (*p* < 0.001); and from 5 (5.21%) to 9 (9.38%) for gout (*p* < 0.004). The number of patient admissions to the hospital for ischemic heart disease decreased significantly from 7 (5.60%) before hemodialysis to 6 (4.80%; Supplementary Table S1) after hemodialysis. Among patients diagnosed with essential hypertension, the probability of admission for hypertension significantly decreased after hemodialysis; specifically, the number of admissions decreased from 99 (20.16%) before hemodialysis to 15 (3.05%) after hemodialysis for hypertension (*p* < 0.001; Supplementary Table S3). Patients diagnosed with anemia exhibited a non-significantly different number of hospital admissions for each of the comorbidities (i.e., diabetes, hypertension, congestive heart failure, ischemic heart disease, cerebrovascular accident, and gout) after they received hemodialysis (Supplementary Table S3). Among patients diagnosed with native atherosclerosis, the probabilities of admission for congestive heart failure and ischemic heart disease increased significantly after hemodialysis. Specifically, the number of admissions increased from 10 (6.21%) before hemodialysis to 25 (15.53%) after hemodialysis for congestive heart failure (*p* = 0.002) and from 14 (11.20%) to 22 (17.60%) for ischemic heart disease (*p* < 0.001; Supplementary Table S4). Among patients diagnosed with urinary tract infection, the probabilities of admission for congestive heart failure and cerebrovascular accident increased significantly after hemodialysis. Specifically, the number of admissions increased from 21 (13.04%) before hemodialysis to 27 (16.77%) after hemodialysis for congestive heart failure (*p* = 0.007) and from 46 (9.22%) to 79 (15.83%) for cerebrovascular accident (*p* = 0.037; Supplementary Table S5). Among patients diagnosed with gastric ulcers who received hemodialysis, the number of admissions decreased from 9 (9.38%) before hemodialysis to 8 (8.33%) after hemodialysis for gout (*p* = 0.049), whereas the number of admissions for cerebrovascular accident remained unchanged at 11 (11.83%; *p* = 0.043; Supplementary Table S6). Among patients diagnosed with pneumonia, the number of admissions to the hospital for congestive heart failure and cerebrovascular accident increased, respectively, from 26 (16.15%) to 34 (21.12%; *p* < 0.001) and from 16 (17.20%) to 22 (23.66%; *p* = 0.004) from before to after hemodialysis (Supplementary Table S7). Among patients diagnosed with reflux esophagitis, the number of admissions increased from 10 (10.75%) before hemodialysis to 15 (16.13%; *p* = 0.037) after hemodialysis for cerebrovascular accident; the number of admissions decreased from 15 (15.63%) to 11 (11.46%) for gout (*p* < 0.001; Supplementary Table S8). Among patients diagnosed with duodenal ulcer, the number of admissions increased from 1 (1.86%) before hemodialysis to 21 (13.04%) after hemodialysis for congestive heart failure (*p* = 0.035; Supplementary Table S9). Among patients diagnosed with hyperkalemia, the number of admissions decreased from 15 (12.00%) to 6 (4.80%) for congestive heart failure (*p* = 0.005; Supplementary Table S10). Finally, among patients diagnosed with bacteremia, the number of hospital admissions increased from 5 (5.21%) before hemodialysis to 11 (11.46%) after hemodialysis for gout (*p* = 0.017; Supplementary Table S11).

## 5. Discussion

External arteriovenous shunts, invented in 1960, facilitate the application of hemodialysis in patients with ESKD. Hemodialysis has had a considerably improved prognosis, having undergone advances over the past 40 years. This improvement is mainly attributable to improved monitoring of medical quality, improved decision-making processes, more streamlined data analysis methods, and new dialysis technologies [8,26,27,28]. The present study on patients with ESKD included patients’ demographic characteristics and medical history in its analysis of the reasons for admission one year before and two years after hemodialysis was received. This study employed a cross-sectional research design in analyzing data on six comorbidities and twelve causes of admission for 592 patients. This study’s sample was female (307) and male (285) patients with approximately equal populations for all age groups. Among the 592 patients, hypertension was the most common comorbidity, which is consistent with findings in the literature; accordingly, hypertension was a prevalent disease in patients receiving hemodialysis.

Demographic statistics from the TSN-KiDiT were analyzed as categorical variables in the McNemar test for cross-sectional analysis. Cross-sectional analysis indicated that age and comorbidities were key variables affecting changes in the type of diagnosis for patients’ hospital admission before and after they started hemodialysis. Additionally, in a subgroup analysis by age, the rate of patients being admitted to the hospital for urinary tract infection, pneumonia, and hyperkalemia was noted to decrease significantly among all age groups. Cardiovascular diseases are considered the most influential risk factor for patients with ESKD during hospitalization. Most patients with ESKD have one or more comorbidities, and almost two-thirds of patients develop ESKD because of diabetes and hypertension; cardiovascular disease is also prevalent comorbidity of ESKD [29]. Accordingly, our study indicated that the six most common comorbidities of ESKD (prevalence rate written in parentheses) were as follows: diabetes (68.75%), hypertension (82.94%), congestive heart failure (27.20%), ischemic heart disease (21.11%), cerebrovascular accident (21.11%), and gout (16.22%). Having multiple comorbidities increases the treatment burden (and, by implication, physical burden) on patients, which leads to higher rates of noncompliance. Similar to another study, this study found that anemia was the second most common comorbidity of ESKD [30]. Among patients who were receiving hemodialysis, the high prevalence of anemia as a comorbidity was attributable to the fact that ESKD increases the risk of anemia [31]. Moreover, the following 11 diseases may also increase the treatment burden and the number of hospital admissions: type 2 diabetes, essential hypertension, anemia, native atherosclerosis, urinary tract infection, gastric ulcer, pneumonia, reflux esophagitis, duodenal ulcer, hyperkalemia, and bacteremia.

Pathogenic comorbidities were more prevalent among older adults [32]. Three related studies have indicated that the incidence of ESKD increases with age. According to the present study’s results, younger participants (aged 65–66 years) were less prone to hospital admission due to atherosclerosis of the native coronary artery, urinary tract infection, or pneumonia. Participants who were admitted to hospitals for hyperkalemia after dialysis had an average age of 64 years. Those who had a urinary tract infection and hyperkalemia before dialysis tended to be older (average age: 70 years old). Finally, participants who developed pneumonia only before or after dialysis were also older (69–70 years on average), and urinary tract infection was more common among older participants.

A clinical epidemiological study reported that uremia in ESKD changed cell and humoral immunity and thus increased patients’ susceptibility to widespread infection. Although a low-grade urinary tract infection does not reduce renal function, the recurrence of said infection can affect the progression of existing renal diseases, thereby causing the renal function to decline [33]. ESKD is a chronic inflammatory condition that increases patients’ susceptibility to widespread infection, and its comorbidities can lead to urinary tract infection due to conditions such as diabetes and incomplete bladder emptying [34].

ESKD is a prevalent condition, particularly in Taiwan, which has the highest incidence of ESKD and has performed the most renal replacement therapies worldwide. Prone to immunodeficiency, patients with ESKD have a higher risk of mortality from infectious diseases than do those without ESKD [5,6]. The high demand for inpatient treatment due to pneumonia and sepsis remains of concern.

Infections constitute a primary factor that drives admission and death among patients receiving dialysis [34]. For patients with ESKD, inpatient treatment for pneumonia is a common practice [35,36]. With regard to the causes of admission, admissions for pneumonia were more common among older patients; specifically, patients who developed pneumonia before or after hemodialysis tended to be older (range: 69–70 years old). Compared with younger patients with ESKD, older patients cannot position themselves or walk on their own, which increases their risk of pneumonia during hospitalization [37]. The present study revealed a significant correlation between pneumonia and ESKD.

Acute nursing care and hospital admissions, which are common among patients receiving dialysis, account for the largest proportion (approximately 40%) of the cost of ESKD treatment [7]. To enhance nursing care quality and resource use efficiency for patients with ESKD, countries should prioritize investigations into the causes or results of these patients’ hospital admissions. One study revealed a positive correlation between the health status and admission rate of patients with ESKD and that the frequency and cause of admission affected their prognosis. On average, a general patient with ESKD is admitted to the hospital twice annually, and the two leading causes for admission are cardiovascular events and infectious diseases [3,38].

ESKD exhibits complex clinical comorbidities, and care for the disease is thus challenging. Such care involves the participation of not only a hemodialysis team but also other medical teams from various specializations (e.g., diabetes, ophthalmology, and neurology); it also involves the provision of health education on chronic kidney disease that is personalized according to the patient’s comorbidities, dialysis type, and lifestyle. Nursing care for ESKD is aimed at improving the patient’s quality of life, arresting the deterioration of kidney functions, and preventing the development of comorbidities. Therefore, such nursing care should be practiced according to the following foci: (1) improve patients’ understanding of the comorbidities of kidney diseases; (2) provide personalized health education (administered by kidney health educators) in accordance with the patient’s comorbidities; (3) communicate the proper health-seeking behaviors and the importance of regular follow-ups to patients to prevent patients from using over-the-counter or folk medicine and to reduce patient drop-outs from dialysis; (4) reduce the admission rate, which is realized by (4a) early contact with patients with ESKD, (4b) cross-team collaboration among medical professionals, and (4c) continual communication with caregivers and patients; (5) provide patients with the appropriate nutritional knowledge for ESKD and understand the dietary compliance of patients to foster appropriate dietary habits in patients. These nursing care foci facilitate comprehensive, continual, professional, and integrative medical care for patients to improve their quality of life.

The present study revealed correlations of comorbidities of ESKD with the duration and cause of hospital admission. It conducted comprehensive data collection on risk factors and comorbidities of ESKD and, with a retrospective design, followed up on the cause of hospital admission of patients with ESKD 1 year before they started dialysis and 2 years after dialysis. The study model involved investigating changes in 100 primary and secondary diagnoses of patients with ESKD before and after dialysis, which could aid in the precise recording of patients’ renal function and prognosis. The data used in this study are of high quality because they were collected from a large-scale renal registration system [39]. The study results highlight the need to control comorbidities associated with ESKD and has implications for clinical practice and research. By integrating test data on patients receiving dialysis, this study provides comprehensive information on patients with ESKD. Investigating patients receiving dialysis, this study offers valuable insights that are conducive to analyzing epidemiological and relevant risk factors among Taiwanese patients receiving dialysis; thus, the findings aid the prevention of renal diseases, enhance the survival of these patients, and reduce the incidence of associated comorbidities.

This study has several limitations. The first lies in its retrospective design and limited sample size. In this study’s retrospective analysis, patients with missing data and records were excluded. Second, the data were collected from the TSN-KiDiT, which lacked clinical indicators critical to the research topic; therefore, this study could not obtain information regarding the severity of certain diseases (e.g., hemodialysis catheter infections, fistula malfunction, blood pressure, and the severities of diabetes and left ventricular hypertrophy). Thus, patients without hemodialysis catheter infections and fistula malfunction data were excluded, which is another limitation of this study. Notably, ICD-10 diagnosis codes adopted after the year 2015 in the National Health Insurance Research Database (NHIRD) have not been validated. Third, ESKD and other comorbidities are defined by ICD-9 codes and may have been misclassified. However, many of the chronic conditions and comorbidities defined by ICD-9 codes have been validated in previous national cohort studies using NHIRD. Additionally, other comorbidities, such as alcohol or substance dependence, have been reported to be associated with ESKD [23], but they were not included in this study’s analysis. Considering the research context of this study, the lack of disease data can lead to results for a disease being underestimated, which can result in the false conclusion that the disease is unrelated to ESKD. Fourth, this study included patients with ESKD who received hemodialysis and had complete medical data 1 year before and 2 years after their hemodialysis. Therefore, patients whose medical data did not conform to the criteria were excluded; this constituted another limitation of this study. Furthermore, this study analyzed only data collected by one hospital. The sample comprised only patients in the hospital who met the inclusion criteria, and some variables may have been overlooked. Because this study focused on the hemodialysis data of only one hospital, this single-center characteristic limits the generalizability of the study results to patients with ESKD with various combinations of comorbidities from other hospitals. Therefore, prospective research involving a larger patient sample size is required to verify the present study’s findings.

## 6. Conclusions

This study investigated the causes of ESKD patients’ admissions to a hospital one year before and two years after they received hemodialysis. This study’s results show that (1) hypertension is the most common comorbidity and (2) cardiovascular diseases are the most influential risk factor for patients with ESKD during hospitalization. Most patients with ESKD exhibited one or more comorbidities, and two-thirds of them developed ESKD because of diabetes and hypertension. With regard to the causes of admission, pneumonia was more prevalent among older patients. Additionally, changes in the diagnosis codes of native atherosclerosis, urinary tract infection, pneumonia, and hyperkalemia were significantly correlated with age. Therefore, when administering comprehensive nursing care and treatment for ESKD, clinicians should not only focus on comorbidities but also consider factors (e.g., age) that can affect patient prognosis.

## Figures and Tables

**Table 1:** Patient demographic characteristics.

Variable	Categories	No. of Patients	Percent
Year of hemodialysis initiation	≤2005	97	16.39
	2006–2010	202	34.12
	2011–2013	133	22.47
	>2013	160	27.03
Sex	Male	285	48.14
	Female	307	51.86
Primary disease category	Kidney disease	144	24.32
	Systemic disease	432	72.97
	Others	16	2.70
Comorbidities Diabetes	No	185	31.25
	Yes	407	68.75
Hypertension	No	101	17.06
	Yes	491	82.94
Congestive heart failure	No	431	72.80
	Yes	161	27.20
Ischemic heart disease	No	467	78.89
	Yes	125	21.11
Cerebrovascular accident	No	499	84.29
	Yes	93	15.71
Gout	No	496	83.78
	Yes	96	16.22

**Table 2:** Hospitalization status before and after hemodialysis.

	Category								
	*No* before and *No* after		*No* before and *Yes* after		*Yes* before and *No* after		*Yes* before and *Yes* after		
	Frequency	Percent	Frequency	Percent	Frequency	Percent	Frequency	Percent	*p* Value
Type 2 diabetes	213	35.98	41	6.93	65	10.98	273	46.11	0.020
Essential hypertension	433	73.14	112	18.92	20	3.38	27	4.56	<0.001
Anemia	316	53.38	124	20.95	76	12.84	76	12.84	<0.001
Native atherosclerosis	423	71.45	28	4.73	74	12.50	67	11.32	<0.001
Urinary tract infection	380	64.19	58	9.80	90	15.20	64	10.81	0.009
Gastric ulcer	443	74.83	34	5.74	91	15.37	24	4.05	<0.001
Pneumonia	384	64.86	58	9.80	113	19.09	37	6.25	<0.001
Reflux esophagitis	451	76.18	40	6.76	68	11.49	33	5.57	0.007
Duodenal ulcer	485	81.93	33	5.57	60	10.14	14	2.36	0.005
Hyperkalemia	466	78.72	69	11.66	41	6.93	16	2.70	0.008
Bacteremia	528	89.19	10	1.69	52	8.78	2	0.34	<0.001

*p* values from McNemar test. *No* before or *No* after—no hospital admission before hemodialysis and no hospital admission after hemodialysis. *No* before and *Yes* after—no hospital admission before hemodialysis and hospital admission after hemodialysis. *Yes* before and *No* after—hospital admission before hemodialysis and no hospital admission after hemodialysis. *Yes* before and *Yes* after—hospital admission before hemodialysis and hospital admission after hemodialysis.

**Table 3:** The relationship between age and the 11 ICD-9-CM codes.

	*No* before and *No* after		*No* before and *Yes* after		*Yes* before and *No* after		*Yes* before and *Yes* after		
Variables	Mean	Standard Error (SE)	Mean	SE	Mean	SE	Mean	SE	*p*
Type 2 diabetes	68.44	13.63	64.02	12.72	66.88	12.03	66.94	10.96	0.082
Essential hypertension	67.76	12.49	63.70	8.96	65.60	12.38	66.52	10.02	0.214
Anemia	67.17	12.33	67.19	11.93	66.14	12.68	74.00	8.72	0.773
Native atherosclerosis	66.37	12.45	70.88	12.52	68.07	9.77	67.60	11.34	0.032 *
Urinary tract infection	65.54	12.14	69.24	12.65	70.79	11.89	70.52	11.40	<0.001 **
Gastric ulcer	66.76	12.36	67.95	11.74	68.29	13.69	69.79	11.11	0.527
Pneumonia	66.03	12.26	69.19	12.44	69.69	11.97	68.65	11.47	0.025 *
Reflux esophagitis	66.96	12.21	67.22	12.75	66.18	12.26	70.82	12.37	0.348
Duodenal ulcer	67.24	12.41	67.38	10.14	66.55	14.12	64.57	12.82	0.863
Hyperkalemia	66.86	12.19	64.07	13.15	70.23	11.71	70.38	13.22	0.040 *
Bacteremia	66.72	12.25	70.67	11.50	71.30	14.06	70.50	27.58	0.101

*p* values from McNemar test. *No* before or *No* after—no hospital admission before hemodialysis and no hospital admission after hemodialysis. *No* before and *Yes* after—no hospital admission before hemodialysis and hospital admission after hemodialysis. *Yes* before and *No* after—hospital admission before hemodialysis and no hospital admission after hemodialysis. *Yes* before and *Yes* after—hospital admission before hemodialysis and hospital admission after hemodialysis. * *p* < 0.05; ** *p* < 0.01.

## Data Availability

This study can confirm this study included a statement regarding data and material availability in the declaration section of my manuscript. The datasets used and/or analyzed during the current study are available from the corresponding author on reasonable request.

## Supplementary Materials

The following supporting information can be downloaded at: https://www.mdpi.com/article/10.3390/ijerph191610253/s1, Table S1. Characteristics of patients diagnosed with type 2 diabetes by group. Table S2. Characteristics of patients diagnosed with essential hypertension by group. Table S3. Characteristics of patients diagnosed with anemia by group. Table S4. Characteristics of patients diagnosed with native atherosclerosis by group. Table S5. Characteristics of patients diagnosed with urinary tract infection by group. Table S6. Characteristics of patients diagnosed with gastric ulcer by group. Table S7. Characteristics of patients diagnosed with pneumonia by group. Table S8. Characteristics of patients diagnosed with reflux esophagitis by group. Table S9. Characteristics of patients diagnosed with duodenal ulcer by group. Table S10. Characteristics of patients diagnosed with hyperkalemia by group. Table S11. Characteristics of patients diagnosed with bacteremia by group.
