# Supplementary material for: Causes of Hospitalization among End-Stage Kidney Disease Cohort before and after Hemodialysis
Source: Int J Environ Res Public Health. 2022 Aug 18;19(16):10253. doi: 10.3390/ijerph191610253 (PMC9408097; doi:10.3390/ijerph191610253)
Supplement: Supplementary file 1 [file ijerph-19-10253-s001.zip › ijerph-1825440-supplementary.pdf]

Supplementary Table S1. Characteristics of patients diagnosed with type 2 diabetes by group. * $p < 0.05$, same for below tables.

		Total		*No* before and *No* after		*No* before and *Yes* after		*Yes* before and *No* after		*Yes* before and *Yes* after		
Variables	Categories	N	%	N	%	N	%	N	%	N	%	P
Year of hemodialysis initiation	<=2005	97	16.39	35	36.08	22	22.68	7	7.22	33	34.02	<.001*
	2006-2010	202	34.12	54	26.73	24	11.88	10	4.95	114	56.44	.
	2011-2013	133	22.47	38	28.57	12	9.02	4	3.01	79	59.40	.
	>2013	160	27.03	86	53.75	7	4.38	20	12.50	47	29.38	.
Sex	1 Male	285	48.14	113	39.65	31	10.88	20	7.02	121	42.46	0.300
	2 Female	307	51.86	100	32.57	34	11.07	21	6.84	152	49.51	.
Primary disease categories	A Kidney disease	144	24.32	126	87.50	9	6.25	1	0.69	8	5.56	<.001*
	B Systemic disease	432	72.97	73	16.90	55	12.73	40	9.26	264	61.11	.
	Others	16	2.70	14	87.50	1	6.25	0	0.00	1	6.25	.
**Comorbidities**												
Diabetes	No	185	31.25	150	81.08	15	8.11	3	1.62	17	9.19	<.001*
	Yes	407	68.75	63	15.48	50	12.29	38	9.34	256	62.90	.
Hypertension	No	101	17.06	52	51.49	13	12.87	6	5.94	30	29.70	0.001*
	Yes	491	82.94	161	32.79	52	10.59	35	7.13	243	49.49	.
Congestive heart failure	No	431	72.80	161	37.35	52	12.06	30	6.96	188	43.62	0.197
	Yes	161	27.20	52	32.30	13	8.07	11	6.83	85	52.80	.
Ischemic heart disease	No	467	78.89	171	36.62	59	12.63	34	7.28	203	43.47	0.023*
	Yes	125	21.11	42	33.60	6	4.80	7	5.60	70	56.00	.
Cerebrovascular accident	No	499	84.29	181	36.27	60	12.02	35	7.01	223	44.69	0.198
	Yes	93	15.71	32	34.41	5	5.38	6	6.45	50	53.76	.
Gout	No	496	83.78	163	32.86	56	11.29	36	7.26	241	48.59	0.004*
	Yes	96	16.22	50	52.08	9	9.38	5	5.21	32	33.33	.

Supplementary Table S2. Characteristics of patients diagnosed with essential hypertension by group

Variables	Categories	Total N	Total %	*No* before and *No* after N	*No* before and *No* after %	*No* before and *Yes* after N	*No* before and *Yes* after %	*Yes* before and *No* after N	*Yes* before and *No* after %	*Yes* before and *Yes* after N	*Yes* before and *Yes* after %	P
Year of hemodialysis initiation	<=2005	97	16.39	55	56.70	8	8.25	15	15.46	19	19.59	<.001*
	2006-2010	202	34.12	142	70.30	3	1.49	51	25.25	6	2.97	.
	2011-2013	133	22.47	104	78.20	5	3.76	23	17.29	1	0.75	.
	>2013	160	27.03	132	82.50	4	2.50	23	14.38	1	0.63	.
Sex	1 Male	285	48.14	208	72.98	5	1.75	61	21.40	11	3.86	0.083
	2 Female	307	51.86	225	73.29	15	4.89	51	16.61	16	5.21	.
Primary disease categories	Kidney disease	144	24.32	123	85.42	2	1.39	15	10.42	4	2.78	<.001*
	Systemic disease	432	72.97	301	69.68	16	3.70	95	21.99	20	4.63	.
	Others	16	2.70	9	56.25	2	12.50	2	12.50	3	18.75	.
**Comorbidities**												
Diabetes	No	185	31.25	148	80.00	3	1.62	25	13.51	9	4.86	0.038
	Yes	407	68.75	285	70.02	17	4.18	87	21.38	18	4.42	.
Hypertension	No	101	17.06	71	70.30	5	4.95	13	12.87	12	11.88	<.001*
	Yes	491	82.94	362	73.73	15	3.05	99	20.16	15	3.05	.
Congestive heart failure	No	431	72.80	317	73.55	15	3.48	79	18.33	20	4.64	0.941
	Yes	161	27.20	116	72.05	5	3.11	33	20.50	7	4.35	.
Ischemic heart disease	No	467	78.89	345	73.88	17	3.64	82	17.56	23	4.93	0.332
	Yes	125	21.11	88	70.40	3	2.40	30	24.00	4	3.20	.
Cerebrovascular accident	No	499	84.29	368	73.75	16	3.21	90	18.04	25	5.01	0.369
	Yes	93	15.71	65	69.89	4	4.30	22	23.66	2	2.15	.
Gout	No	496	83.78	358	72.18	19	3.83	95	19.15	24	4.84	0.416
	Yes	96	16.22	75	78.13	1	1.04	17	17.71	3	3.13	.

Supplementary Table S3. Characteristics of patients diagnosed with anemia by group

Variables	Categories	Total N	Total %	*No* before and *No* after N	*No* before and *No* after %	*No* before and *Yes* after N	*No* before and *Yes* after %	*Yes* before and *No* after N	*Yes* before and *No* after %	*Yes* before and *Yes* after N	*Yes* before and *Yes* after %	P
Year of hemodialysis initiation	<=2005	97	16.39	84	86.60	11	11.34	1	1.03	1	1.03	0.005*
	2006-2010	202	34.12	166	82.18	26	12.87	9	4.46	1	0.50	.
	2011-2013	133	22.47	114	85.71	9	6.77	9	6.77	1	0.75	.
	>2013	160	27.03	149	93.13	2	1.25	9	5.63	0	0.00	.
Sex	1 Male	285	48.14	244	85.61	19	6.67	19	6.67	3	1.05	0.028*
	2 Female	307	51.86	269	87.62	29	9.45	9	2.93	0	0.00	.
Primary disease categories	A Kidney disease	144	24.32	124	86.11	13	9.03	7	4.86	0	0.00	0.660
	B Systemic disease	432	72.97	377	87.27	32	7.41	20	4.63	3	0.69	.
	Others	16	2.70	12	75.00	3	18.75	1	6.25	0	0.00	.
**Comorbidities**												
Diabetes	No	185	31.25	154	83.24	21	11.35	10	5.41	0	0.00	0.140
	Yes	407	68.75	359	88.21	27	6.63	18	4.42	3	0.74	.
Hypertension	No	101	17.06	86	85.15	8	7.92	7	6.93	0	0.00	0.593
	Yes	491	82.94	427	86.97	40	8.15	21	4.28	3	0.61	.
Congestive heart failure	No	431	72.80	375	87.01	35	8.12	18	4.18	3	0.70	0.538
	Yes	161	27.20	138	85.71	13	8.07	10	6.21	0	0.00	.
Ischemic heart disease	No	467	78.89	407	87.15	36	7.71	21	4.50	3	0.64	0.670
	Yes	125	21.11	106	84.80	12	9.60	7	5.60	0	0.00	.
Cerebrovascular accident	No	499	84.29	436	87.37	40	8.02	22	4.41	1	0.20	0.079
	Yes	93	15.71	77	82.80	8	8.60	6	6.45	2	2.15	.
Gout	No	496	83.78	435	87.70	38	7.66	20	4.03	3	0.60	0.182
	Yes	96	16.22	78	81.25	10	10.42	8	8.33	0	0.00	.

Supplementary Table S4. Characteristics of patients diagnosed with native atherosclerosis by group

Variables	Categories	Total N	Total %	*No* before and *No* after N	*No* before and *No* after %	*No* before and *Yes* after N	*No* before and *Yes* after %	*Yes* before and *No* after N	*Yes* before and *No* after %	*Yes* before and *Yes* after N	*Yes* before and *Yes* after %	P
Year of hemodialysis initiation	<=2005	97	16.39	73	75.26	15	15.46	2	2.06	7	7.22	0.045*
	2006-2010	202	34.12	134	66.34	26	12.87	9	4.46	33	16.34	.
	2011-2013	133	22.47	89	66.92	20	15.04	9	6.77	15	11.28	.
	>2013	160	27.03	127	79.38	13	8.13	8	5.00	12	7.50	.
Sex	1 Male	285	48.14	196	68.77	30	10.53	19	6.67	40	14.04	0.017*
	2 Female	307	51.86	227	73.94	44	14.33	9	2.93	27	8.79	.
Primary disease categories	A Kidney disease	144	24.32	109	75.69	12	8.33	5	3.47	18	12.50	0.189
	B Systemic disease	432	72.97	299	69.21	61	14.12	23	5.32	49	11.34	.
	Others	16	2.70	15	93.75	1	6.25	0	0.00	0	0.00	.
**Comorbidities**												
Diabetes	No	185	31.25	139	75.14	17	9.19	9	4.86	20	10.81	0.401
	Yes	407	68.75	284	69.78	57	14.00	19	4.67	47	11.55	.
Hypertension	No	101	17.06	80	79.21	8	7.92	7	6.93	6	5.94	0.059
	Yes	491	82.94	343	69.86	66	13.44	21	4.28	61	12.42	.
Congestive heart failure	No	431	72.80	326	75.64	49	11.37	18	4.18	38	8.82	0.002*
	Yes	161	27.20	97	60.25	25	15.53	10	6.21	29	18.01	.
Ischemic heart disease	No	467	78.89	383	82.01	52	11.13	14	3.00	18	3.85	<.001*
	Yes	125	21.11	40	32.00	22	17.60	14	11.20	49	39.20	.
Cerebrovascular accident	No	499	84.29	363	72.75	61	12.22	21	4.21	54	10.82	0.327
	Yes	93	15.71	60	64.52	13	13.98	7	7.53	13	13.98	.
Gout	No	496	83.78	355	71.57	62	12.50	21	4.23	58	11.69	0.577
	Yes	96	16.22	68	70.83	12	12.50	7	7.29	9	9.38	.

Supplementary Table S5. Characteristics of patients diagnosed with urinary tract infection by group

Variables	Categories	Total		*No* before and *No* after		*No* before and *Yes* after		*Yes* before and *No* after		*Yes* before and *Yes* after		
		N	%	N	%	N	%	N	%	N	%	P
Year of hemodialysis initiation	<=2005	97	16.39	61	62.89	16	16.49	9	9.28	11	11.34	<.001*
	2006-2010	202	34.12	119	58.91	42	20.79	20	9.90	21	10.40	.
	2011-2013	133	22.47	75	56.39	25	18.80	12	9.02	21	15.79	.
	>2013	160	27.03	125	78.13	7	4.38	17	10.63	11	6.88	.
Sex	1 Male	285	48.14	221	77.54	30	10.53	19	6.67	15	5.26	<.001*
	2 Female	307	51.86	159	51.79	60	19.54	39	12.70	49	15.96	.
Primary disease categories	A Kidney disease	144	24.32	89	61.81	24	16.67	17	11.81	14	9.72	0.795
	B Systemic disease	432	72.97	281	65.05	62	14.35	40	9.26	49	11.34	.
	Others	16	2.70	10	62.50	4	25.00	1	6.25	1	6.25	.
**Comorbidities**												
Diabetes	No	185	31.25	122	65.95	27	14.59	19	10.27	17	9.19	0.823
	Yes	407	68.75	258	63.39	63	15.48	39	9.58	47	11.55	.
Hypertension	No	101	17.06	62	61.39	19	18.81	8	7.92	12	11.88	0.624
	Yes	491	82.94	318	64.77	71	14.46	50	10.18	52	10.59	.
Congestive heart failure	No	431	72.80	293	67.98	63	14.62	37	8.58	38	8.82	0.007*
	Yes	161	27.20	87	54.04	27	16.77	21	13.04	26	16.15	.
Ischemic heart disease	No	467	78.89	296	63.38	71	15.20	49	10.49	51	10.92	0.720
	Yes	125	21.11	84	67.20	19	15.20	9	7.20	13	10.40	.
Cerebrovascular accident	No	499	84.29	327	65.53	79	15.83	46	9.22	47	9.42	0.037*
	Yes	93	15.71	53	56.99	11	11.83	12	12.90	17	18.28	.
Gout	No	496	83.78	325	65.52	73	14.72	44	8.87	54	10.89	0.260
	Yes	96	16.22	55	57.29	17	17.71	14	14.58	10	10.42	.

Supplementary Table S6. Characteristics of patients diagnosed with gastric ulcer by group

Variables	Categories	Total		*No* before and *No* after		*No* before and *Yes* after		*Yes* before and *No* after		*Yes* before and *Yes* after		
		N	%	N	%	N	%	N	%	N	%	P
Year of hemodialysis initiation	<=2005	97	16.39	72	74.23	21	21.65	2	2.06	2	2.06	<.001*
	2006-2010	202	34.12	132	65.35	45	22.28	12	5.94	13	6.44	.
	2011-2013	133	22.47	97	72.93	18	13.53	11	8.27	7	5.26	.
	>2013	160	27.03	142	88.75	7	4.38	9	5.63	2	1.25	.
Sex	1 Male	285	48.14	224	78.60	39	13.68	13	4.56	9	3.16	0.214
	2 Female	307	51.86	219	71.34	52	16.94	21	6.84	15	4.89	.
Primary disease categories	A Kidney disease	144	24.32	113	78.47	16	11.11	7	4.86	8	5.56	0.577
	B Systemic disease	432	72.97	317	73.38	73	16.90	26	6.02	16	3.70	.
	Others	16	2.70	13	81.25	2	12.50	1	6.25	0	0.00	.
**Comorbidities**												
Diabetes	No	185	31.25	142	76.76	26	14.05	8	4.32	9	4.86	0.611
	Yes	407	68.75	301	73.96	65	15.97	26	6.39	15	3.69	.
Hypertension	No	101	17.06	76	75.25	17	16.83	4	3.96	4	3.96	0.838
	Yes	491	82.94	367	74.75	74	15.07	30	6.11	20	4.07	.
Congestive heart failure	No	431	72.80	332	77.03	63	14.62	23	5.34	13	3.02	0.099
	Yes	161	27.20	111	68.94	28	17.39	11	6.83	11	6.83	.
Ischemic heart disease	No	467	78.89	356	76.23	70	14.99	22	4.71	19	4.07	0.180
	Yes	125	21.11	87	69.60	21	16.80	12	9.60	5	4.00	.
Cerebrovascular accident	No	499	84.29	376	75.35	80	16.03	23	4.61	20	4.01	0.043*
	Yes	93	15.71	67	72.04	11	11.83	11	11.83	4	4.30	.
Gout	No	496	83.78	366	73.79	83	16.73	25	5.04	22	4.44	0.049*
	Yes	96	16.22	77	80.21	8	8.33	9	9.38	2	2.08	.

Supplementary Table S7. Characteristics of patients diagnosed with pneumonia by group

Variables	Categories	Total		*No* before and *No* after		*No* before and *Yes* after		*Yes* before and *No* after		*Yes* before and *Yes* after		
		N	%	N	%	N	%	N	%	N	%	P
Year of hemodialysis initiation	<=2005	97	16.39	77	79.38	16	16.49	3	3.09	1	1.03	<.001*
	2006-2010	202	34.12	113	55.94	48	23.76	26	12.87	15	7.43	.
	2011-2013	133	22.47	66	49.62	38	28.57	14	10.53	15	11.28	.
	>2013	160	27.03	128	80.00	11	6.88	15	9.38	6	3.75	.
Sex	1 Male	285	48.14	183	64.21	51	17.89	29	10.18	22	7.72	0.489
	2 Female	307	51.86	201	65.47	62	20.20	29	9.45	15	4.89	.
Primary disease categories	A Kidney disease	144	24.32	94	65.28	31	21.53	11	7.64	8	5.56	0.705
	B Systemic disease	432	72.97	278	64.35	79	18.29	47	10.88	28	6.48	.
	Others	16	2.70	12	75.00	3	18.75	0	0.00	1	6.25	.
**Comorbidities**												
Diabetes	No	185	31.25	120	64.86	40	21.62	13	7.03	12	6.49	0.386
	Yes	407	68.75	264	64.86	73	17.94	45	11.06	25	6.14	.
Hypertension	No	101	17.06	68	67.33	18	17.82	7	6.93	8	7.92	0.619
	Yes	491	82.94	316	64.36	95	19.35	51	10.39	29	5.91	.
Congestive heart failure	No	431	72.80	298	69.14	79	18.33	32	7.42	22	5.10	<.001*
	Yes	161	27.20	86	53.42	34	21.12	26	16.15	15	9.32	.
Ischemic heart disease	No	467	78.89	306	65.52	91	19.49	41	8.78	29	6.21	0.444
	Yes	125	21.11	78	62.40	22	17.60	17	13.60	8	6.40	.
Cerebrovascular accident	No	499	84.29	338	67.74	91	18.24	42	8.42	28	5.61	0.004*
	Yes	93	15.71	46	49.46	22	23.66	16	17.20	9	9.68	.
Gout	No	496	83.78	329	66.33	93	18.75	46	9.27	28	5.65	0.279
	Yes	96	16.22	55	57.29	20	20.83	12	12.50	9	9.38	.

Supplementary Table S8. Characteristics of patients diagnosed with reflux esophagitis by group

Variables	Categories	Total		*No* before and *No* after		*No* before and *Yes* after		*Yes* before and *No* after		*Yes* before and *Yes* after		
		N	%	N	%	N	%	N	%	N	%	P
Year of hemodialysis initiation	<=2005	97	16.39	90	92.78	6	6.19	1	1.03	0	0.00	<.001*
	2006-2010	202	34.12	163	80.69	23	11.39	11	5.45	5	2.48	.
	2011-2013	133	22.47	81	60.90	25	18.80	11	8.27	16	12.03	.
	>2013	160	27.03	117	73.13	14	8.75	17	10.63	12	7.50	.
Sex	1 Male	285	48.14	217	76.14	27	9.47	22	7.72	19	6.67	0.276
	2 Female	307	51.86	234	76.22	41	13.36	18	5.86	14	4.56	.
Primary disease categories	A Kidney disease	144	24.32	109	75.69	14	9.72	12	8.33	9	6.25	0.749
	B Systemic disease	432	72.97	328	75.93	52	12.04	28	6.48	24	5.56	.
	Others	16	2.70	14	87.50	2	12.50	0	0.00	0	0.00	.
**Comorbidities**												
Diabetes	No	185	31.25	140	75.68	19	10.27	16	8.65	10	5.41	0.616
	Yes	407	68.75	311	76.41	49	12.04	24	5.90	23	5.65	.
Hypertension	No	101	17.06	77	76.24	9	8.91	9	8.91	6	5.94	0.666
	Yes	491	82.94	374	76.17	59	12.02	31	6.31	27	5.50	.
Congestive heart failure	No	431	72.80	328	76.10	49	11.37	31	7.19	23	5.34	0.890
	Yes	161	27.20	123	76.40	19	11.80	9	5.59	10	6.21	.
Ischemic heart disease	No	467	78.89	355	76.02	54	11.56	29	6.21	29	6.21	0.457
	Yes	125	21.11	96	76.80	14	11.20	11	8.80	4	3.20	.
Cerebrovascular accident	No	499	84.29	391	78.36	53	10.62	30	6.01	25	5.01	0.037*
	Yes	93	15.71	60	64.52	15	16.13	10	10.75	8	8.60	.
Gout	No	496	83.78	389	78.43	57	11.49	25	5.04	25	5.04	<.001*
	Yes	96	16.22	62	64.58	11	11.46	15	15.63	8	8.33	.

Supplementary Table S9. Characteristics of patients diagnosed with duodenal ulcer by group

Variables	Categories	Total		*No* before and *No* after		*No* before and *Yes* after		*Yes* before and *No* after		*Yes* before and *Yes* after		
		N	%	N	%	N	%	N	%	N	%	P
Year of hemodialysis initiation	<=2005	97	16.39	83	85.57	12	12.37	1	1.03	1	1.03	<.001*
	2006-2010	202	34.12	155	76.73	25	12.38	14	6.93	8	3.96	.
	2011-2013	133	22.47	98	73.68	18	13.53	12	9.02	5	3.76	.
	>2013	160	27.03	149	93.13	5	3.13	6	3.75	0	0.00	.
Sex	1 Male	285	48.14	242	84.91	23	8.07	15	5.26	5	1.75	0.276
	2 Female	307	51.86	243	79.15	37	12.05	18	5.86	9	2.93	.
Primary disease categories	A Kidney disease	144	24.32	114	79.17	14	9.72	11	7.64	5	3.47	0.771
	B Systemic disease	432	72.97	357	82.64	45	10.42	21	4.86	9	2.08	.
	Others	16	2.70	14	87.50	1	6.25	1	6.25	0	0.00	.
**Comorbidities**												
Diabetes	No	185	31.25	147	79.46	19	10.27	14	7.57	5	2.70	0.522
	Yes	407	68.75	338	83.05	41	10.07	19	4.67	9	2.21	.
Hypertension	No	101	17.06	83	82.18	10	9.90	6	5.94	2	1.98	0.990
	Yes	491	82.94	402	81.87	50	10.18	27	5.50	12	2.44	.
Congestive heart failure	No	431	72.80	350	81.21	39	9.05	30	6.96	12	2.78	0.035*
	Yes	161	27.20	135	83.85	21	13.04	3	1.86	2	1.24	.
Ischemic heart disease	No	467	78.89	385	82.44	46	9.85	25	5.35	11	2.36	0.931
	Yes	125	21.11	100	80.00	14	11.20	8	6.40	3	2.40	.
Cerebrovascular accident	No	499	84.29	413	82.77	50	10.02	25	5.01	11	2.20	0.480
	Yes	93	15.71	72	77.42	10	10.75	8	8.60	3	3.23	.
Gout	No	496	83.78	403	81.25	53	10.69	26	5.24	14	2.82	0.221
	Yes	96	16.22	82	85.42	7	7.29	7	7.29	0	0.00	.

Supplementary Table S10. Characteristics of patients diagnosed with hyperkalemia by group

Variables	Categories	Total		*No* before and *No* after		*No* before and *Yes* after		*Yes* before and *No* after		*Yes* before and *Yes* after		P
		N	%	N	%	N	%	N	%	N	%	
Year of hemodialysis initiation	<=2005	97	16.39	81	83.51	7	7.22	8	8.25	1	1.03	0.135
	2006-2010	202	34.12	157	77.72	18	8.91	23	11.39	4	1.98	.
	2011-2013	133	22.47	95	71.43	9	6.77	24	18.05	5	3.76	.
	>2013	160	27.03	133	83.13	7	4.38	14	8.75	6	3.75	.
Sex	1 Male	285	48.14	238	83.51	20	7.02	22	7.72	5	1.75	0.013*
	2 Female	307	51.86	228	74.27	21	6.84	47	15.31	11	3.58	.
Primary disease categories	A Kidney disease	144	24.32	114	79.17	10	6.94	19	13.19	1	0.69	0.541
	B Systemic disease	432	72.97	339	78.47	29	6.71	49	11.34	15	3.47	.
	Others	16	2.70	13	81.25	2	12.50	1	6.25	0	0.00	.
**Comorbidities**												
Diabetes	No	185	31.25	145	78.38	16	8.65	22	11.89	2	1.08	0.285
	Yes	407	68.75	321	78.87	25	6.14	47	11.55	14	3.44	.
Hypertension	No	101	17.06	80	79.21	11	10.89	8	7.92	2	1.98	0.216
	Yes	491	82.94	386	78.62	30	6.11	61	12.42	14	2.85	.
Congestive heart failure	No	431	72.80	348	80.74	30	6.96	45	10.44	8	1.86	0.071
	Yes	161	27.20	118	73.29	11	6.83	24	14.91	8	4.97	.
Ischemic heart disease	No	467	78.89	371	79.44	35	7.49	54	11.56	7	1.50	0.005*
	Yes	125	21.11	95	76.00	6	4.80	15	12.00	9	7.20	.
Cerebrovascular accident	No	499	84.29	396	79.36	34	6.81	57	11.42	12	2.40	0.702
	Yes	93	15.71	70	75.27	7	7.53	12	12.90	4	4.30	.
Gout	No	496	83.78	397	80.04	29	5.85	56	11.29	14	2.82	0.096
	Yes	96	16.22	69	71.88	12	12.50	13	13.54	2	2.08	.

Supplementary Table S11. Characteristics of patients diagnosed with bacteremia by group

Variables	Categories	Total N	Total %	*No* before and *No* after N	*No* before and *No* after %	*No* before and *Yes* after N	*No* before and *Yes* after %	*Yes* before and *No* after N	*Yes* before and *No* after %	*Yes* before and *Yes* after N	*Yes* before and *Yes* after %	P
Year of hemodialysis initiation	<=2005	97	16.39	93	95.88	4	4.12	0	0.00	0	0.00	0.023*
	2006-2010	202	34.12	181	89.60	16	7.92	4	1.98	1	0.50	.
	2011-2013	133	22.47	107	80.45	22	16.54	4	3.01	0	0.00	.
	>2013	160	27.03	147	91.88	10	6.25	2	1.25	1	0.63	.
Sex	1 Male	285	48.14	254	89.12	25	8.77	4	1.40	2	0.70	0.490
	2 Female	307	51.86	274	89.25	27	8.79	6	1.95	0	0.00	.
Primary disease categories	A Kidney disease	144	24.32	129	89.58	10	6.94	4	2.78	1	0.69	0.786
	B Systemic disease	432	72.97	385	89.12	40	9.26	6	1.39	1	0.23	.
	Others	16	2.70	14	87.50	2	12.50	0	0.00	0	0.00	.
**Comorbidities**												
Diabetes	No	185	31.25	166	89.73	13	7.03	4	2.16	2	1.08	0.127
	Yes	407	68.75	362	88.94	39	9.58	6	1.47	0	0.00	.
Hypertension	No	101	17.06	91	90.10	8	7.92	1	0.99	1	0.99	0.572
	Yes	491	82.94	437	89.00	44	8.96	9	1.83	1	0.20	.
Congestive heart failure	No	431	72.80	392	90.95	30	6.96	7	1.62	2	0.46	0.062
	Yes	161	27.20	136	84.47	22	13.66	3	1.86	0	0.00	.
Ischemic heart disease	No	467	78.89	415	88.87	43	9.21	7	1.50	2	0.43	0.685
	Yes	125	21.11	113	90.40	9	7.20	3	2.40	0	0.00	.
Cerebrovascular accident	No	499	84.29	447	89.58	43	8.62	7	1.40	2	0.40	0.558
	Yes	93	15.71	81	87.10	9	9.68	3	3.23	0	0.00	.
Gout	No	496	83.78	448	90.32	41	8.27	5	1.01	2	0.40	0.017*
	Yes	96	16.22	80	83.33	11	11.46	5	5.21	0	0.00	.
